# Comparative Analysis of Saponins from Different *Phytolaccaceae* Species and Their Antiproliferative Activities

**DOI:** 10.3390/molecules22071077

**Published:** 2017-06-29

**Authors:** Flora Didii Saleri, Guilin Chen, Xun Li, Mingquan Guo

**Affiliations:** 1 Key Laboratory of Plant Germplasm Enhancement and Specialty Agriculture, Wuhan Botanical Garden, Chinese Academy of Sciences, Wuhan 430074, China; didiiflora@gmail.com (F.D.S.); cjl1652009@163.com (G.C.); lixunyiyi@126.com (X.L.); 2 University of Chinese Academy of Sciences, Beijing 100049, China; 3 Sino-Africa Joint Research Center, Chinese Academy of Sciences, Wuhan 430074, China

**Keywords:** *Phytolacca acinosa*, *Phytolacca americana*, triterpenoid saponin, ultrasound-assisted extraction, LC-MS, antiproliferative activities

## Abstract

The quality and the efficacy of herbal medicine are of great concern especially with the increase in their global use. Medicinal plants of different species or collected from different geographical regions have shown variations in both their contents and pharmacological activities due to the differences in the environmental conditions of the collected sites. In this study, roots of *Phytolacca acinosa* found in different provinces in south China (Sichuan and Shandong) and a species of *Phytolacca americana* were investigated. To ensure a maximum yield of the major compounds, the extraction method and conditions were optimized. The preeminent method of extraction in this analysis was determined to be the ultrasound-assisted method with specific conditions as follows: ethanol-H_2_O (1:1, *v*/*v*), with a solvent: sample ratio of 1:8, and extraction was performed 3 times, each for 30 min. Under these conditions, samples from the different regions varied both in quantity and quality via the LC-MS analysis. A total of 60 triterpenoid saponins were detected within the three samples, among which 22 were identified as common in the three samples. The amounts of these common triterpenoid saponin identified varied across the samples. Moreover, the analysis led to the detection of some novel compounds that have not yet been reported in this family, while other compounds differ in their fragmentation pathways compared to previous literature. To further divulge the correlations between the bioactivities in these three samples and the quantity and quality of their bioactive components, a cytotoxic analysis was thus carried out with two cancer cell lines, and SGC-7901 and Hep G2, which evidently showed remarkable differences in their anti-proliferative activities with respect to the IC_50_ value. Samples of *P. acinosa* from Sichuan showed higher values in both cell lines (27.20 ± 1.60 and 25.59 ± 1.63 µg/mL) compared to those of Shandong and *P. americana*. For the first time, analysis and comparison of both interspecies and of different species in this family were carried out. This study will significantly contribute to the quality insurance of herbal medicine, especially in the Phytolaccaceae family.

## 1. Introduction

Phytolaccaceae species are perennial herbs that are distributed globally with different species native to a particular place. These plant species have been used both traditionally and in modern times as herbal medicines [1]. The chemical constituents of the species in this family have shown a number of bioactive activities including antibacterial [2,3], antifungal [4,5,6,7,8,9], antimalarial [10,11], molluscicidal properties [12,13], and anti-obesity [14], potential behavioral and pro-oxidant effects [15], antitumor activities [16], among others. The compounds responsible for the above activities are reported to be triterpenoid saponins [2,3,4,5,6,7,8,9,10,11,12,13].

Recent research on a particular species of this family (*P. acinosa*) showed that, a major compound, escutentoside A, was used against tumor, hyperplasia of mammary glands, and endometriosis in clinics and has shown potent therapeutic effects [17]. Its derivatives exhibited inhibitory activities towards COX-2 and hemolysis [18]. A study by André Straus et al. showed that the cultured root of *P. acinosa* contained saponin [19]. However, there is a challenge in ensuring the efficacy of the active compounds due to the variability in the quality and the quantity of the natural chemical compounds. On the other hand, the secondary metabolic constituents play important roles in plant adaptation, that is, they are triggered and varied so that plants may adapt to specific conditions (water stress, heat, and light intensity, herbivore and microbial attack) [20]. Thus, environmental conditions are one of the major factors that have been suggested to influence the variations [20,21,22,23]. Studies have shown that plants collected from different geographies have shown variations in terms of quantity and also the types of compounds present [24].

**Figure 1:**
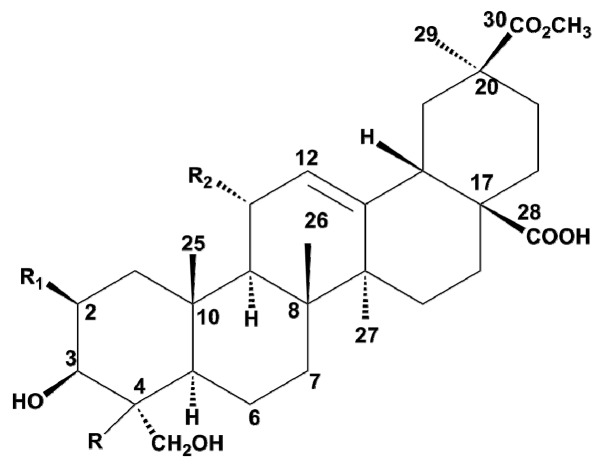
Pentacyclic structures of aglycones described in Phytolaccaceae family.

Several studies on this plant family have focused mainly on separation and isolation, structural characterization and the investigations of their pharmacological and biological properties. Recent research by Guillermo et al. successfully identified about 30 phytolacca saponins using HPLC techniques coupled with ESI-MS in both positive and negative modes, and NMR for further structural elucidation. Moreover, the research illustrated the corresponding types of aglycones described in this family as shown in Figure 1 [25,26]. Research that involves fingerprinting has proven that this technique is of great potential in hierarchal classification of plants in the same family [27], showing relationship between plants in classification. It also assists in determining the constituency of herbal medicine and quality assessment. Moreover, this technique coupled with other hyphenated chromatographic approaches has enabled identification of novel compounds and also enhances identification of minor chemical metabolites [27,28]. In the present work, saponins in Phytolacceae collected from different geographical locations and species have been qualitatively and quantitatively analyzed, and their differences in anti-proliferative activity were also studied in order to reveal the correlations between them. However, there is a considerable time lag in this area of study on the Phytolacceae family. It is generally believed that changes in the active compositions of the plant material could affect its therapeutic activity. Thus, it is of key importance to conduct a thorough analysis on the saponins in these species and their corresponding biological activities, which may help ensure the efficacy and safety of these species for medicinal use. In addition, this work may aid in better understanding and improving the quality control of medicinal plants.

## 2. Results and Discussion

### 2.1. Optimization of Extraction Method

High recovery and enrichment of active ingredients from medicinal plants are mainly influenced during the extraction steps with heat or agitation as the major focus in most of the extraction methods. The fundamental parameters influencing the production of a good quality extract are the type of solvent used, solvent: sample ratio, extraction method, the number of extraction times and the duration of extraction, among others, depending on the method in use [29]. To optimize the extraction method, 20 g of ground root material was extracted using two methods; ultrasound assisted method and heat reflux method. The concentrations of total saponin (mg)/gram of the extract from both methods were analysed using a UV-vis spectrophotometer. The ultrasound assisted method was found to have a concentration of total saponin of 38.87 mg/g of the plant extract compared to that of heat reflux method (36.04 mg/g of extract in reference to the standard curve of oleonolic acid). Further optimization revealed the optimized conditions for extraction are as follows: ethanol-H_2_O (1:1, *v*/*v*), 30 min, 3 extractions and a sample ratio of 1:8. In addition, the method was preferred since it required a short period of time and low temperatures, thus avoiding damage to extracts and also any loss of volatile components. The agitation in this method facilitates swelling and hydration, thus bulging in the pores, resulting in the penetration of the solvent into the cellular membrane and release of the components into the solvent [30,31].

### 2.2. Qualitative and Quantitative Analysis of Saponins

Not much is known as to how the environmental factors may possibly influence saponin content. However, with the help of modern techniques such as liquid chromatography-mass spectrometry (LC-MS), an inventive insight into the significant changes of numerous secondary metabolites, particularly the triterpenoid saponins, may be detected and identified for the purpose of fingerprinting comparisons of plant samples from different locations or species. Furthermore, this technique has proven to be efficient in the identification and structure elucidation of triterpenoid saponins, which are known to cause difficulties in their analysis due to their occurrence in the complex mixture with very minute amounts together with many other plant secondary metabolites and their complicated structural formation with slight differences from each other, a high polarity, as well as the isobaric nature [32,33].

**Figure 2:**
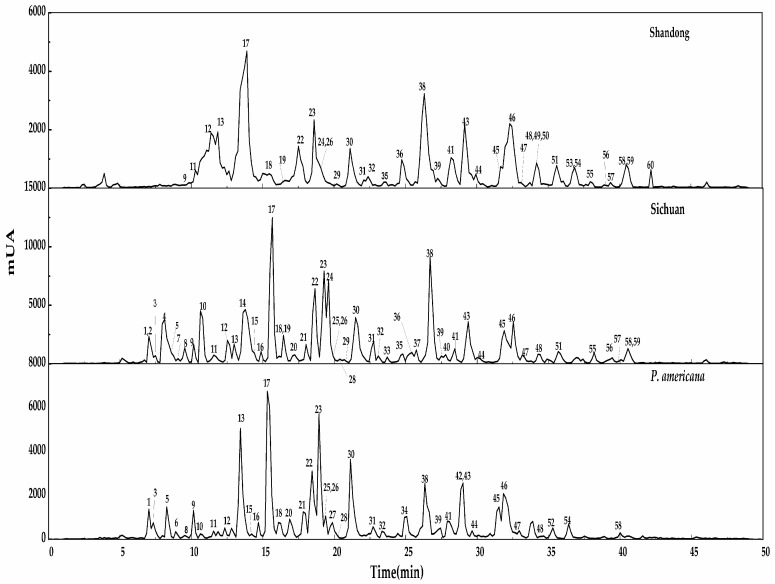
LC-MS profiles of three samples from Shandong, Sichuan and *P. americana.*

After the three samples roots were extracted under the optimized conditions, Liquid-Liquid extraction with petroleum ether followed by saturated 1-butanol was carried out. Column chromatography (AB-8 macroporous resins) was then carried out on the concentrated saturated *n*-butanol fraction with ethanol-H_2_O (7:3, *v*/*v*). The enriched saponins fraction from the AB-8 column of *P. acinosa* (two regions) and the *P. americana* species were analysed in parallel for the identification of the different compounds present. Figure 2 shows the LC-MS chromatogram of the three root samples of Chinese *P. acinosa* (Shandong and Sichuan) and *P. americana* species. LC-MS used for qualitative analysis of the saponins was coupled with ElectroSpray ionization (ESI) at a mass range of *m*/*z* 500–1500. It was performed in the negative mode, yielding intense deprotonated molecular ions [M − H]^−^. LC-MS analysis carried out revealed the fragmentation pathway and the resultant different aglycone formed, which contributed to identification. It also yielded the qualitative outcome that facilitated the comparison among the samples, as shown in Table 1. Moreover, the spectra enabled the investigation of the areas of common peaks detected for quantitative comparisons as shown in Table 2. First, optimizations of the different conditions including the gradient elution, the sample concentration, the mass range and time were performed, and triplicate tests for each sample were carried out under optimal conditions. A total of 60 triterpenoid saponins were identified, among which 39 compounds were detected in the *P. americana* sample, 37 in the Shandong and 50 from Sichuan province among the mentioned detected compounds; a number of them were detected for the first time.

The compounds identified were further structurally described by the loss of the sugar moiety (Hexose or Pentose loss). Other minor losses included H_2_O, CO_2_, molecules of 44 Da, 60 Da among others. The retention time of previous research majorly on this plant family and others that have been reported to have similar aglycones also contributed to the structural descriptions, i.e., compounds of the same molecular weight compared to the retention time of already identified compounds. Fragments formed in the MS^n^ were also compared with previous research so as to yield a similar structural description and pattern of loss of the sugar moiety.

### 2.3. Structural Identifications of Saponins Using LC-ESI-MS/MS Analysis

Table 1 shows the triterpenoid saponins that have been detected and their proposed compositions. The fragmentation description was through the study of how the component fragments and the resultant aglycones formed after their fragmentation, which also assisted in identification. For the isomeric saponin, retention time from previous studies and fragments formed facilitated the differentiation between them. The general structural formation of all saponins identified shows the attachment of one or more sugar chains to the aglycone. The saponin may either be mono- desmosidic, i.e., a single sugar chain, typically attached at C-3, or bidesmosidic, i.e., two sugar chains attached to C-3 and C-28.

#### 2.3.1. Identification of the Peaks Based on Aglycone

The aglycone detected assisted in identification of the compounds and was further used in categorizing the compound into 7 clusters: phytolaccagenic acid, 11α-hydroxyphytolaccagenin, 11α-methoxyphytolaccagenin, 11-oxo-phytolaccagenin, phytolaccagenin, 2-oxo-phytolaccagenin acid or serjanic acid aglycone. More research may be done in future to distinguish the contribution of aglycone in the quantitative and qualitative correlations, especially in the bioactivity analysis.

##### Identification of Peaks with Phytolaccagenic Acid Aglycone

Nineteen compounds were tentatively identified with phytolaccagenic acid as their aglycone at *m*/*z* 515 despite differences in the fragmentation through the loss of sugar [−162 and/or −132 Da], H_2_O, CO_2_ among other losses. Peaks 4, 10, 15, 18, 19, 23, 25, 26, 32, 33, 34, 36, 39, 44, 45, 51, 52, 54 and 60 were placed in this group, and their fragmentations are shown in Table 1. The fragmentations of Peaks 4, 10, 19, 23, 33 and 60 showed some similarity in their MS/MS fragmentation. Peak 4 at *m*/*z* 973 showed an abundant fragment ion at *m*/*z* 973 [M − H − 162 Da − 162 Da]^−^, followed by a subsequent loss of a pentose sugar [−132 Da]. Peak 19 was attentively identified as an isomer of peak 4 and their identifications were comparable to previous research [34]. The subsequent loss of ions at peaks 10 and 23 at *m*/*z* 811 led to them being placed in this group. The continuous fragmentation shows the loss in *m*/*z* 811 of peak 10, which led to fragments at *m*/*z* 691, 649, 631, 515 comparable to those of peak 23, which had an additional fragment of *m*/*z* 795. Peaks 33 and 60, identified as isomers at *m*/*z* 649, were also in this category. Their fragmentations were similar to those of fragments at peaks 4, 10 and 23. Peaks 15, 18, 25, 26, 44, 51 and 54 were identified with some similarity in their fragments formed at *m*/*z* 809, 663, 647, 629 and 515, which were as a result of the loss of at least more than one hexose sugar [−162 Da] and a pentose sugar [−132 Da]. Peaks 25 and 51 were tentatively identified as isomers, and their fragmentation was compared to those of other studies [25,26]. Moreover, peak 51 had an extra fragment of *m*/*z* 791, which was as a result of the loss of H_2_O after the loss of the first pentose sugar from the parent ion. The abundance of the ion at *m*/*z* 809 and resultant fragment ions at peaks 18, 32 and 54 resulted in the same molecular weight. However, their structural conformation differed according to the MS^n^ and the retention time as compared to previous research [25,38]. As for Peaks 15, 26 and 44, their identification was also compared to those of previous studies [25,39]. Their fragmentation showed abundance in the fragment ions formed at the aglycone level. In regards to peak 45, the MS/MS showed loss as follows: *m*/*z* 839 [M − H − 162 Da − 132 Da − 30 Da]^−^, which led to the *m*/*z* 515 ion as an abundant fragment aglycone. Peaks 36 and 52 showed good abundance in their ions in the MS spectra. For peak 36, MS/MS fragmentation confirmed a successive loss of two sugar residues at *m*/*z* 853 [M − H − 162 Da − 132 Da − 30 Da]^−^ in successive fragmentation, which led to only one fragment ion *m*/*z* 515. The cleavage on peak 52 resulted in only one fragment at *m*/*z* 515. As for peak 34 at *m*/*z* 653, the abundant fragment ion was identified as aglycone, which enabled us to categorise it in this group. Its pattern of fragmentation revealed a successive loss of a pentose sugar [−132 Da], which resulted in ions of *m*/*z* 571 and 529. The last one in this category is peak 39, *m*/*z* 915, whose fragmentation led to six fragment ions at *m*/*z* 779, 677, 633, 603 and 529 due to subsequent loss of the sugar moiety, resulting in an abundant aglycone of *m*/*z* 515.

##### Identification of Peaks with 2-oxo-Phytolaccagenin Acid Aglycone

**Figure 3:**
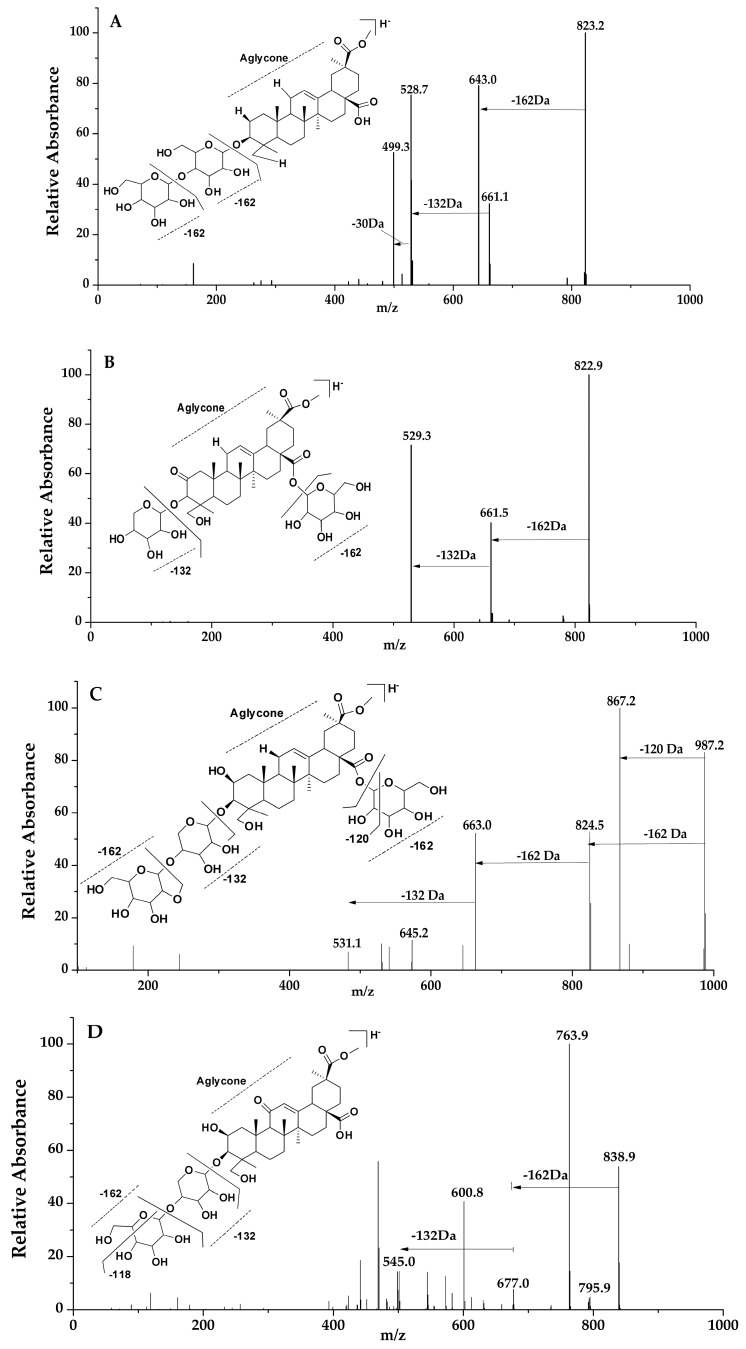
LC-MS spectra and fragmentation pathway; peaks 41, 43, 12 and 5 (**A**–**D**) respectively.

In this group, the saponin had a 2-oxo-Phytolacagenin acid aglycone formed of *m*/*z* 529, and peaks of 11, 16, 21, 31, 41, 43 and 57 were placed in this category. Based on the MS/MS data, peaks of 11, 31, 41 and 42 of molecular weight *m*/*z* 823 were tentatively identified and termed to be isomers due to their fragmentation pattern and abundance. However, in reference to the previous research by Guillermo 2009, the compound showed a structural difference, and identification was compared to that of the retention times of previous studies [25]. For peaks 11 and 31, the fragments ion of these compounds were formed by the loss of a hexose residue [−162 Da] and a pentose residue [−132 Da] from the main compound and were termed as isomers due to their similarity in the LC-MS MS^n^ spectra fragments. Peaks 41 and 43 were compared with retention times of previous studies [25]. For peak 41, Figure 3A, two fragments were formed by the loss of a hexose [−162 Da] and a pentose [−132 Da] residue from the abundant peak at *m*/*z* 823. Fragmentation pathway of peak 43, Figure 3B, was dissimilar to that of peak 41 although they had the same molecular mass in that the first cleavage resulted in the loss C_2_H_4_O [−44 Da] followed by the loss of the remaining hexose sugar fragment [−118 Da] forming a fragment ion of *m*/*z* 661; further fragments were formed as a result of cleavage, losing [−44 Da] and finally, a loss of [−88 Da] from the parent ion; as a result, a pentose sugar was lost. Another isomer identified in this group was that of peaks 16 and 21 with molecular ion of *m*/*z* 867. Only one cleavage occurred according to the MS/MS data, and it produced an ion of *m*/*z* 529, meaning possible a loss of a hexose sugars [−162 Da] and a pentose [−132 Da] and [−44 Da] molecule. Peak 57 represented a molecular ion at *m*/*z* 721 with fragment ions at *m*/*z* 721 [M − H − 60 Da]^−^ and *m*/*z* 721 [M − H − 60 Da − 132 Da]^−^.

##### Identification of Peaks with Phytolaccagenin Aglycone

Triterpenoid saponins identified in the category of Phytolaccagenin acid as the aglycone, characterized by a fragment ion at *m*/*z* 531, included peaks 6, 7, 9, 12, 13, 14, 17, 24, 29, 37, 46, 58 and 59. Similarities in fragmentations were tentatively identified at peaks 7, 12, 13, 14, 29 and 46. Fragment ions at *m*/*z* 825, 663, 645 and 531 were common for these peaks. The fragments were as a result of a subsequent loss of a hexose sugar [−162 Da] followed by a loss of a pentose sugar [−132 Da] residue from the identified peak. Peaks 13 and 14 of *m*/*z* 825 were identified as isomers as described in previous studies [25]. Their MS/MS data show a loss of a hexose [−162 Da] and pentose [−132 Da] sugar. For peak 12 at *m*/*z* 987, fragmentation shown in Figure 3C had a hexose [−162 Da], a residue more than that of peaks 13 and 14, while peak 7 at *m*/*z* 1149 had two more hexose residues in addition to peaks 13 and 14; their identification was compared to those of previous research based on the retention time [26,37]. Its fragmentation clearly showed the loss of three hexoses [−162 Da] and a pentose sugar [−132 Da]. As for peak 29 at *m*/*z* 663, it showed only one fragment at *m*/*z* 531, indicating one pentose [−132 Da] residue. Peak 6 also had one loss of [−102 Da], while peak 9 at *m*/*z* 1031 and peak 17 at *m*/*z* 869 showed a similar fragmentation pattern by losing *m*/*z* 1031 [M − H − 338 Da − 162 Da]^−^ and *m*/*z* 869[M − H − 338 Da]^−^, respectively, which may imply a loss of a hexose residues [−162 Da] and a pentose sugar [−132 Da] and a [−44 Da] molecule. As for peak 24, the MS/MS data revealed the loss of a hexose sugar [−162 Da] and a [−132 Da] pentose sugar. Peak 37 showed a loss of more than four sugar residues to form the aglycone at *m*/*z* 531. In regard to peak 58 at *m*/*z* 723, MS/MS data showed the loss of [−60 Da], forming a fragment at *m*/*z* 663, followed by the loss of a pentose residue, *m*/*z* 723 [M − H − 60 Da − 132 Da]^−^. Peak 59 fragments were as a result of a cleavage, losing four hexose sugars [−162 Da] and H_2_O, while the second cleavage led to the loss of a pentose sugar [−132 Da].

##### Identification of Peaks with 11α-Hydroxyphytolaccagenin Aglycone

The fragmentation pathway of compounds at peaks 1, 2, 3, 27, 47, 48, 49, 50 and 55 led to the formation of compounds with 11α-hydroxyphytolaccagenin of *m*/*z* 547 as the abundant aglycone. The peaks identified by MS/MS analysis in this group showed some similarity in their fragments. Isomeric compounds were identified, i.e., peaks 1, 27 and 48. For peak 1, fragmentation depicted a subsequent loss of the hexose [−162 Da] residue, followed by a loss of a pentose sugar [−132 Da] moiety resulting in several fragments. For peaks 27 and 48, the fragments formed were due to the loss of hexose [−162 Da] and pentose [−132 Da] residues from the original compound, thus resulting in only two fragments. Peaks 47and 55 were also tentatively identified as isomers at *m*/*z* 1045, and their fragmentation patterns were comparable. The fragment ions detected were at *m*/*z* 915, 799, 663 and 547. Data of peaks 2, 49 and 50 MS/MS showed the presence of fragments that were similar to those of peak 1, i.e., *m*/*z* 841, formed by the loss of a hexose [−162 Da] residue of peak 2. Peak 49 fragments were similar to those of peak 1 with an additional fragment due to an additional [−60 Da] molecule. Lastly, peak 3 presented a fragment in one cleavage, as in *m*/*z* 883 [M − H − 336]^−^, followed by a second cleavage in *m*/*z* 547 [M − H − 336 − 48]^−^ as the abundant fragment. In summary, in terms of analysis of molecule loss, two hexose sugars and a [−60 Da] molecule were lost.

##### Identification of Peaks with Serjanic Acid Aglycone

According to MS/MS data and the pathways of fragmentation, peak 28 was placed in this category since the abundant ion detected was *m*/*z* 499. Peak 28 detected the loss of a hexose [−162 Da] residue and a pentose [−132 Da] residue in three cleaveages.

##### Identification of Peaks with 11-oxo-Phytolaccagenin Aglycone

The resultant aglycone at *m*/*z* 545 identified in compounds of peaks 5, 22, 30, 38, 42, 53 and 56 led to them being categorized in this group. Peak 5, i.e., Figure 3D, and peak 30 yielded fragment ions comparable to those that have been previously studied as 3-[Xyl-(1→4)-Glc]-28-Glc-11-oxo-Phytolaccagenin [35]. Peak 22 at *m*/*z* 963 [M − H]^−^ yielded four major fragments at *m*/*z* 933, 591, 561 and 545. For peak 38, its fragmentation pathway showed the loss of mainly a total of two hexose sugars [−162 Da], which resulted in five fragments. In regards to peak 53, three fragments were formed by the subsequent loss of [−60 Da] and a pentose sugar [−132 Da] molecule. Peak 56 formed fragments at *m*/*z* 779, 617 and 545, which were compared to fragments that have been previously studied [36]. Peak 42’s fragments resembled those of peak 56 starting at *m*/*z* 779 [M − H]^−^, implying one sugar less than at peak 56.

##### Identification of Peaks with 11α-Methoxyphytolaccagenin Aglycone

The final group in the classification was characterized by ions at *m*/*z* 562. Four peaks were tentatively identified according to the literature of previous studies using their fragmentation pathways. Peaks 8, 20, 35 and 40 were detected at *m*/*z* 855, although their structures differ according to their retention times. The characteristic fragments formed were at *m*/*z* 779, 679, 663, 617 and 531, comparable to the study conducted by Guillermo et al., 2009 [25,39].

### 2.4. Assessment of the Identified Triterpenoid Saponins in the Three Samples

**Figure 4:**
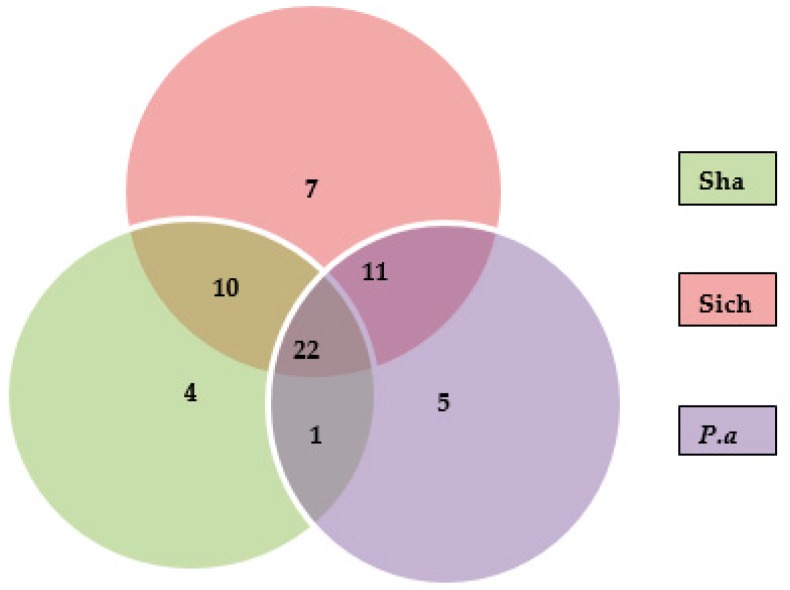
Summary of the number of saponins identified in/among the samples.

Based on the MS/MS data analysis, a total of 60 compounds were identified as shown in Table 1. Among the compounds identified, 39 were detected in *P. americana*, 37 in the Shandong sample while, 50 compounds were present in the Sichuan samples of *P. acinosa*. Moreover, a total of 22 compounds of peaks (9, 11, 12, 13, 17, 18, 22, 23, 26, 30, 31, 32, 38, 39, 41, 43, 44, 45, 46, 47, 48 and 58) were summarized as common among the three samples. Meanwhile, some peaks were found in two samples; peaks (1, 3, 5, 6, 10, 15, 16, 20, 21, 25 and 28) were detected in the *P. americana* sample and the Sichuan sample, peaks (8, 19, 24, 29, 35, 36, 51, 55, 56, 57 and 59) were detected in Shandong and Sichuan sample while peak (54) was detected in *P. americana* and Shandong samples. Peaks identified in individual samples may result in differences, mainly in the quality and quantity of each sample; these include peaks (6, 27, 34, 42 and 52) for *P. americana* sample. Peaks (50, 49, 53 and 60) were identified only in Shandong, while (2, 4, 7, 14, 33, 37 and 40) were identified only in the Sichuan sample. Figure 4 summarizes Section 2.4, showing the number of triterpenoid saponins identified in individual samples and those that were found common between and/or among the samples.

Based on the chromatogram in Figure 2, the semi-quantitative analysis was used to investigate the three samples. Table 2 gives the % relative areas obtained from the spectra which give the relative quantities in the common compounds in the three samples. The areas of the peaks showed contrasts across the three samples. The percentage relative area was calculated as: Relative area of common peaks (%) = (Area of the selected common peaks/summation of all the areas of the detected peaks) × 100%.

### 2.5. Antiproliferative Activity Assay

**Figure 5:**
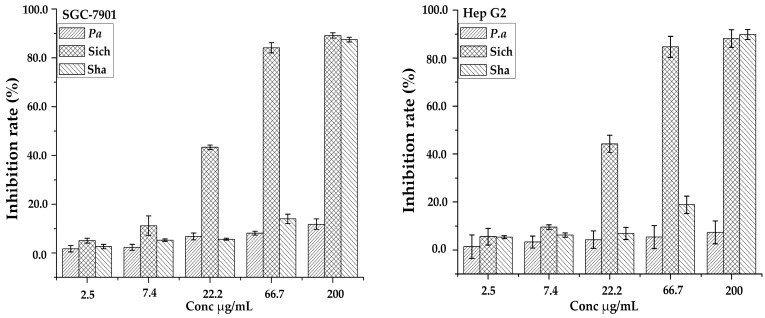
Inhibition rates of three samples against two cell lines (SCG-7901 and Hep G2).

Saponins have a broad range of biological activities that have drawn attention from pre-historic times; one of them being antitumor activity has recently been a major concern worldwide. The triterpenoid saponins have potential antitumor activities and exerted their inhibition effects via different assays in vitro. The ethanol-H_2_O (7:3, *v*/*v*) fractions of the three samples from the AB-8 column were evaluated for anti-proliferative activities against two human tumor cell lines of gastric carcinoma (SCG-7901) and colorectal carcinoma (Hep G2) as shown in Figure 5. It was found that sample Sichuan had higher antiproliferative activities against SGC-7901 and Hep G2 cells with IC_50_ values of 27.20 ± 1.60 and 25.59 ± 1.63 µg/mL, respectively. The results clearly demonstrated the variation in their bioactivity between the samples from different regions and in different species across the cell lines. This analysis also revealed that the inhibition of growth of cancer cells is dependent on the dosage of the extract. It may be suggested that the quantity of the common compounds in Table 2 detected in each sample may have contributed to their variability in bioactivity. Hence further study may be carried out on them. Furthermore, peaks unique to a specific sample may also have influenced the variability in the inhibition rate.

## 3. Experimental Section

### 3.1. Chemicals Reagents

Oleonolic saponin standard and HPLC-grade solvents (acetonitrile and acetic acid) were purchased from Sigma-Aldrich Corp. (Shanghai, China). HPLC-grade water was obtained using a Milli-Q System (Millipore, Billerica, MA, USA). Other chemicals of analytical grade were obtained from China Medicine Group and Shanghai Chemical Reagent Corp. (Shanghai, China). Millipore membranes (0.22 µm) were purchased from Jinteng Experiment Equipment Corp. (Tianjin, China). AB-8 macroporous resin was purchased from an industrial chemical company affiliated with Nan Kai University (Tianjin, China).

### 3.2. Biological Material

*Phytolacca acinosa* samples were collected from different regions of China: Sichuan, Shandong, and one sample of *Phytolacca americana* was collected at Wuhan Botanical Garden during the flowering season. Authentication and identification of the specimens were assisted by the taxonomist (Guangwan Hu) of Key Laboratory of Plant Germplasm Enhancement and Agriculture Specialty (Wuhan Botanical Garden), Chinese Academy of Sciences. A voucher specimen (No. 0032, 0033 and 0034) for *P. acinosa* (Sichuan and Shandong) and *P. americana* was deposited in the herbarium of the Key Laboratory. For the preparation of all extracts, 50 g of each sample was oven-dried at 50 °C, ground to a powder and stored in air-tight desiccators until use.

### 3.3. Evaluation of Extraction Method

Two commonly used methods were investigated, i.e., heat reflux extraction and ultrasonic method, according to Yi et al., 2007 with few modifications; 20 g of powdered sample was extracted with 160 mL of ethanol-H_2_O (7:3, *v*/*v*), reflux was performed at 80 °C for 1.5 h, 3 times while the ultrasonic method was carried out at room temperature for 30 min, 3 times. Further optimization of individual factors was also carried out.

### 3.4. Colorimetric Method for Quantification Analysis

The determination of the percentage content of total triterpenoid saponin was performed as described by Zhang et al., 2001 [41] with minor modifications. The standard curve was developed with concentrations of 0.0, 0.1, 0.2, 0.4, 0.6 and 0.8 mL oleanolic acid standard solution precisely measured in a 10 mL test tube. The solvent was evaporated in a water bath and 0.4 mL newly mixed 5% (*w*/*v*) vanillin-acetic acid solution and 0.8 mL perchloric acid were added, mixed and incubated at 70 °C for 15 min. The tubes were taken out and cooled under running water for 2 min, and acetic acid was added to make up a total volume of 5 mL. The absorbance rate was measured with a UV/Vis spectrophotometer at 548 nm. To determine the triterpenoid saponin in the samples, 0.2 mL of the extracted root sample was used following the method as described.

### 3.5. Extraction of Triterpenoid Saponins

The powdered material was extracted using and ultrasonic method with optimized conditions; (ethanol-H_2_O (1:1, *v*/*v*) at a sample ratio of 1:8; the sample was extracted for 30 min, 3 times). The filtrates were concentrated to dryness in a rotary evaporator under reduced pressure at 40 °C. Liquid-liquid extraction was performed with petroleum ether and water (1:1, *v*/*v*), and enrichment of the saponin was performed with saturated 1-butanol with water (1:1, *v*/*v*) three times. The 1-butanol fraction was concentrated and re-dissolved in a small amount of methanol and then added to a portion <5 g of the AB-8 resins before being loaded into the column. Column chromatography was performed as per Lifeng et al., 2011 with modifications. A macroporous resin AB-8 (50 g) was loaded on the glass column. Elution was first performed with 250 mL H_2_O, followed by 250 mL of ethanol-H_2_O (7:3, *v*/*v*), resulting in two fractions. The ethanol-H_2_O (7:3, *v*/*v*) as the expected fraction was concentrated to dryness and was dissolved in 3 mL methanol-H_2_O (3:2, *v*/*v*); then, 0.5 mL was filtered using Millipore membranes (0.22 µm) and transferred to an autosampler vial for LC/ESI-MS analysis [42].

### 3.6. Untargeted Metabolite Profiling by Liquid Chromatography-Mass Spectrometry

LC separations were optimized and eventually performed on a Thermo Accela 600 HPLC (Thermo Fisher Scientific, Waltham, MA, USA) system using a reverse-phase analytical column (150 × 4.6 mm, 3 µm; BDS C-18). The mobile phase (water/acetonitrile) comprised 0.1% acetic acid (A) and acetonitrile (B). An aliquot of a 10 µL sample solution was injected into the HPLC system, and the linear eluting gradient was as follow: 0–15 min at 25–31% B, 15–45 min at 31–40% B, and 45–50 min at 40–50% B at a flow rate of 0.4 mL/min, and the column temperature was maintained at 30 °C. For the ESI-MS/MS experiment, a Thermo Accela 600 LC system coupled to a TSQ Quantum Access MAX mass spectrometer (Thermo Fisher Scientific, San Jose, CA, USA) equipped with an ESI source operating in Auto-MS^n^ mode to obtain fragmentation in the negative mode MS conditions were set as follows: source voltage, 3.0 kV; cone voltage, 40.0 V; desolvation temperature, 350 °C; capillary temperature, 250 °C; nebulizing gas flow rate, 6.0 L/min; sheath gas (N_2_) pressure, 40 arb; Aux gas (N_2_) pressure, 10 arb; collision energy (CE), 10 V; collision energy grad (CE grad), 0.035 V/m. Mass spectra data were obtained with the full-scan mode for *m*/*z* in the range from 500 to 1500, and the nine most abundant ions were selected for further MS^2^ spectra. All data acquisition and analysis were performed using the Thermo Xcalibur ChemStation (Thermo Fisher Scientific, San Jose, CA, USA).

### 3.7. Antiproliferation Assays in Vitro

#### 3.7.1. Cell Culture

The two human cancer cell lines of SGC-7901 (gastric carcinoma) and Hep G2 (hepatoma carcinoma) were obtained from the China Center for Type Culture Collection (CCTCC) (Wuhan, China). Cells were routinely grown in culture medium containing Dulbecco’s modified Eagle’s medium (DMEM) medium supplemented with 10% fetal bovine serum (FBS), glutamine (2 mM) and 1% penicillin (100 U/mL)-streptomycin (100 µg/mL). Furthermore, the cell lines were sub-cultured twice a week, and incubated in a humidified atmosphere with 5% CO_2_ and 90% relative humidity (RH) under 37 °C. The number of living cells was assessed using a hematocytometer and phase-contrast microscopy. Cells with over 80% confluence (growth phase) were used for the following cell antiproliferation assay [43].

#### 3.7.2. Cytotoxic Analysis Using Sulforhodamine B (SRB) Growth Assay

The protein-staining sulforhodamine B (SRB) microculture colorimetric assay with some modification was used in the estimation of the antiproliferative activities against SGC-7901 and Hep G2 [44]. Research has shown that the SRB assay shows high sensitivity to total cellular protein content and linearity to cell density and is thus used for in vitro anticancer evaluation at the National Cancer Institute (Bethesda, MD, USA) [45]. Briefly, a 100 µL cell suspension of the trypsinized monolayer cells in DMEM medium was seeded into 96-well plates with a density of 3.5 × 10^4^ cells per well. After incubation at 37 °C in 5% CO_2_ and 90% relative humidity for 24 h to resume exponential growth and stabilization, the culture medium were then carefully detached, and an aliquot of 100 µL ethanol H_2_O (7:3, *v*/*v*) AB-8 fraction was added into each well in the plates. The three samples were first dissolved in DMSO and further diluted with the medium to a final DMSO content less than 0.1%, which was innocuous to cell growth and proliferation. After incubation for another 72 h, cells were fixed with 50 µL 10% cold (4 °C) trichloroacetic acid (TCA) for 30 min at 4 °C. The supernatants were washed out with deionized water five times and air-dried at room temperature. The dried plates were stained with 100 µL 4 mg/mL SRB in 1% acetic acid solution for 30 min at room temperature. The plates were then washed five times with 1% acetic acid to remove the unbound SRB and then air-dried overnight. The protein-bound SRB was dissolved with 150 µL of 10 mM Tris base (pH 10.5), and the plates were left on a gyratory shaker for 10 min. The complete medium with less than 0.1% DMSO was used as the control. The IC_50_ values were determined with a 96-well plate reader (Tecan, Männedorf, Switzerland) at a wavelength of 490 nm. The % cell inhibition was determined using the following equation:

% cell inhibition = (mean OD control − mean OD sample)/mean OD control × 100%

where ODC and ODS are the OD values of controls and the three samples, respectively. Analysis of each sample was performed in triplicate, and results are expressed as the mean ± SD.

## 4. Conclusions

In summary, chromatographic fingerprinting analysis has attracted great attention, and such analysis has proven to be a useful tool for quality control of herbal plants. Significant differences in the three different samples collected from different regions and one from a different species were found. The quantity and quality analysis of Phytolaccaceae were determined by first evaluating the most effective method between ultrasonic and heat reflux extraction method. It was found that the ultrasonic method was more efficient in extracting triterpenoid saponins from this family with conditions as follows; ethanol-H_2_O (1:1, *v*/*v*), with a solvent: sample ratio of 1:8 and 3 times subsequent extraction, each performed for 30 min. LC-ESI/MS was also optimized, and the results were used in the comparison of the peaks (compounds) present and not present among the samples (qualitative analysis); a total of 60 compounds as in Table 1 were identified and some were termed as novel compounds. Additionally, the LC-ESI/MS indicated the profiles of the abundances (quantitative analysis) of metabolites in the three medicinal Phytolacca samples, yielding the % relative area for the compounds detected using the analysis software. In this study, the identification of the target compounds was based on the retention time compared to other analysis and fragmentation pathways, especially with regard to the linkages of their sugar residues and lastly the resultant aglycone. For the anti-proliferative analysis, the samples showed promising potential activity against the two cells with increase in dosage. From the results, one can deduce that despite more compounds detected in *P. americana*, its inhibition activity was lower than the sample that had less compounds detected at all concentrations. The concentrations of the active compounds that have the cytotoxicity activity were higher in the sample (Sichuan) with an IC_50_ of 27.20 ± 1.60 and 25.59 ± 1.63 µg/mL in the cells SGC-7901 and Hep G2, respectively. This may be as a result of variation in the common peaks as shown in Table 2, i.e., despite the quality of compounds detected, the quantities of the compounds in the sample also play a major role in its bioactivity. Shandong and Sichuan sample, though, are of the same species, and there was variability in their quality and quantity as evident in the analysis carried out. This proves that the environmental conditions play a part in influencing the chemical constituents in plants. Further analysis may be carried out to investigate the specific compounds responsible for the anti-proliferative activity. This work provides, for the first time, a fingerprinting study on this family and has clearly shown the correlation between the environmental factors and how they affect the bioactive constituents in plants. Moreover, it has also emphasized the chemical difference between species of the same family, therefore contributing to the prominence of quality assurance and efficacy of the medicinal plant.

## Figures and Tables

**Aglycone**	**MW**	**R**	**R1**	**R2**
Serjanic acid (**1**)	500	H	H	H
Phytolaccagenic acid (**2**)	516	OH	H	H
2-oxo-Phytolacagenin acid (**3**)	530	OH	=O	H
Phytolaccagenin (**4**)	532	OH	OH	H
11-oxo-Phytolacagenin (**5**)	546	OH	OH	=O
11α-Methoxyphytolaccagenin (**6**)	562	OH	OH	OCH_3_
11α-Hydroxyphytolaccagenin (**7**)	548	OH	OH	OH

**Table 1:** Identification of triterpenoid saponins corresponding to Figure 2 LC-ESI-MS/MS chromatogram of three samples.

Peak	RT (min)	*m*/*z*	Fragmentation [M − H]^−^	*P. a*	Sha	Sich	Tentative Structural Elucidation	Ref.
1	7.02	841	841, 779, 679, 617, 547, 529	√	*	√	3-[Xyl]-28-Glc-11α-hydroxyphytolaccagenin	[25]
2	7.34	1003	1003, 841, 679,661, 547, 529	*	*	√	3-[Xyl-(1→4)-Glc]-28-Glc-11α-hydroxyphytolaccagenin	[25]
3	7.36	883	883, 547, 499	√	*	√	Unknown	
4	8.12	973	973, 811, 795, 779, 649, 631, 517	*	*	√	3-[Glc-(1→3)-Ara]-28-Glc-phytolaccagenic acid	[34]
5	8.30	839	839, 795, 763, 677, 633, 601, 545,515	√	*	√	3-[Xyl-(1→4)-Glc)]-28-Glc-11-oxophytolaccagenin	[35,36]
6	9.00	633	633, 531	√	*	*	Unknown	
7	9.06	1149	1149, 987, 825, 663, 645, 531	*	*	√	3-[Xyl-(1→4)-Glc-(1→2)-Glc]-28-Glc-phytolaccagenin	[25,26]
8	9.53	855	855, 779, 693,679, 663, 617, 531	√	*	√	3-[Xyl]-28-Glc-11α-methoxyphytolaccagenin	[36]
9	10.16	1031	1031, 693, 531	√	√	√	Unknown	
10	10.71	811	811, 691, 649, 631, 515, 499	√	*	√	3-[Ara]-28-Glc-phytolaccagenic acid	[34]
11	11.55	823	823, 661, 643, 531, 529	√	√	√	3-[Xyl-(1→4)-Glc]-2-oxophytolaccagenic acid	[25,36]
12	12.42	987	987, 825, 663, 645, 531	√	√	√	3-[Xyl-(1→4)-Glc]-28-Glc-phytolaccagenin	[26]
13	12.90	825	825, 663, 645, 627, 531	√	√	√	3-[Xyl]-28-Glc-phytolaccagenin	[26,37]
14	13.77	825	825, 663, 645, 531	*	*	√	Isomer ^13^	
15	14.12	1133	1133, 971, 809, 663, 647, 515	√	*	√	3-[Xyl-(1→4)-Glc-(1→2)-Glc]-28-Glc-phytolaccagenic acid	[25,36]
16	14.74	867	867, 529	√	*	√	Unknown	
17	14.85	869	869, 531	√	√	√	Unknown	
18	16.35	809	809, 647, 629, 515	√	√	√	3-[Xyl]-28-Glc-phytolaccagenic acid	[38]
19	16.57	973	973, 811, 795, 779, 649, 631, 515,	*	√	√	Isomer ^4^	
20	16.98	855	855, 779, 693,679, 663, 617, 531	√	*	√	Isomer ^8^	
21	18.00	867	867, 529	√	*	√	Isomer ^16^	
22	18.20	963	963, 933, 591, 569, 545, 529	√	√	√	unknown	
23	18.93	811	811, 795, 693, 649, 631, 515	√	√	√	Isomer ^10^	
24	19.08	885	885, 663, 617, 531	*	√	√	Unknown	
25	19.61	955	955, 809, 647,629, 515	√	*	√	3-[Xyl-(1→4)-Glc-(1→2)-Rha]-phytolaccagenic acid	[25]
26	19.93	1117	1117, 955, 809, 647, 629,515	√	√	√	3-[LRha-(1→2)-Glc-(1→2)-Rha]-28-Glc-phytolaccagenic acid	[25,26]
27	19.97	841	841, 679, 547	√	*	*	Isomer ^1^	
28	20.39	795	795, 633, 531, 501	√	*	√	3-[Ara]-28-Glc-serjanic	[33]
29	20.44	663	663, 531	*	√	√	3-[Xyl]-phytolaccagenin	[25]
30	21.25	839	839, 677, 545	√	√	√	Isomer ^5^	
31	22.62	823	823, 661, 643, 531, 529	√	√	√	Isomer ^11^	
32	22.79	809	809, 647, 529,515	√	√	√	Isomer ^18^	
33	23.43	649	649, 517	*	*	√	Unknown	
34	24.25	653	653, 571, 529, 515	√	*	*	Unknown	
35	24.86	855	855, 779, 693, 663, 531	*	√	√	3-[Xyl-(1→4)-Glc]-11α-methoxyphytolaccagenin	[25]
36	25.02	853	853, 775,630,515	*	√	√	Unknown	
37	26.21	1355	1355, 1163, 677, 661, 531,515	*	*	√	Unknown	
38	26.45	945	945, 915, 707, 591, 561, 545	√	√	√	Unknown	
39	27.02	915	915, 779, 677, 633, 603, 529, 515	√	√	√	Unknown	
40	27.80	855	855, 779, 693, 663, 531	*	*	√	Isomer ^35^	
41	28.23	823	823, 661, 529	√	√	√	3-[Xyl]-28-2-oxophytolaccagenic acid	[25]
42	28.99	779	779, 617, 545	√	*	*	Unknown	
43	29.14	823	823, 779, 661, 631, 617, 529	√	√	√	3-[Xyl-(1→4)-Glc]-2-oxophytolaccagenic acid	[25]
44	29.89	971	971, 809, 647, 515	√	√	√	3-[Glc-(1→3)-LAra]-28-Glc-phytolaccagenic acid	[39]
45	31.69	839	839, 677, 545, 515	√	√	√	3-[Glc-(1→4)-Glc]-phytolaccagenic acid	[25]
46	32.33	825	825, 663, 645, 531, 499	√	√	√	3-[Xyl-(1→4)-Glc]-phytolaccagenin	[25]
47	33.06	1045	1045, 915, 799, 663, 547	√	√	√	Unknown	
48	33.68	841	841, 679, 547, 515	√	√	√	Isomer ^1^	
49	33.70	901	901, 841, 547	*	√	*	Unknown	
50	33.72	1171	1171, 841, 679, 547	*	√	*	Unknown	
51	35.56	955	955, 809, 791, 647, 629, 515	*	√	√	Isomer ^25^	
52	35.62	835	835, 515	√	*	*	Unknown	
53	36.63	737	737, 677, 665, 545	*	√	*	Unknown	
54	36.86	809	809, 647, 629, 515	√	√	*	3-[Xyl-(1→4)-Glc]-phytolaccagenic acid	[25]
55	38.10	1045	1045, 915, 799, 709, 663, 547	*	√	√	Isomer ^47^	
56	39.05	925	925, 779, 617, 545	*	√	√	3-[Glc-(1→4)-Rha]-11-oxo-phytolaccagenin	[40]
57	39.36	721	721, 661, 529	*	√	√	Unknown	
58	40.69	723	723, 663, 531	√	√	√	Unknown	
59	41.01	1329	1329, 663, 562, 531	*	√	√	Unknown	
60	44.68	649	649, 631, 517	*	√	*	Isomer ^33^	

Abbreviations: *P.a*, *Phytolacca americana*; Sha, Shandong sample; Sich, Sichuan samples of *P. acinosa.* √ = Compound detected; * = Compound not detected. Isomer ^(No.)^ = Numbers in parentheses represent the peak corresponding to isomers. Glc (glucose), Gal (galactose), Ara (arabinose), Xyl (xylose), Rha (Rhamnose).

**Table 2:** Semi-quantitative analysis of the % relative areas of common peaks.

% Relative Peak Area of Common Peaks				
Peak	*m*/*z*	*P. a*	Sha	Sich
9	1031	1.97	0.43	1.00
11	823	0.42	0.32	1.26
12	987	0.58	7.20	1.98
13	825	8.12	7.20	1.26
17	869	11.48	18.84	14.37
18	809	1.59	2.55	1.46
22	963	5.21	4.39	8.52
23	811	7.20	3.24	6.86
26	1117	0.59	0.13	0.70
30	839	6.23	3.02	6.21
31	823	1.25	0.34	1.92
32	809	0.64	0.70	0.62
38	945	4.14	9.23	9.01
39	915	1.21	0.75	0.47
41	823	2.13	2.94	1.29
43	823	2.53	2.44	4.52
44	971	0.38	0.77	0.74
45	839	2.73	3.11	5.49
46	825	5.89	7.28	3.69
47	1045	1.56	0.37	1.02
48	841	0.79	5.58	1.24
58	723	0.47	1.01	1.96
